# Emerging New Targets in Systemic Therapy for Malignant Pleural Mesothelioma

**DOI:** 10.3390/cancers16071252

**Published:** 2024-03-22

**Authors:** Karen M. Yun, Lyudmila Bazhenova

**Affiliations:** Division of Hematology-Oncology, Moores Cancer Center at UC San Diego Health, La Jolla, CA 92093, USA; lbazhenova@health.ucsd.edu

**Keywords:** BAP1, malignant pleural mesothelioma, mesothelin, NF2, targeted therapy

## Abstract

**Simple Summary:**

Malignant pleural mesothelioma is an aggressive cancer that is not surgically resectable for the majority of patients diagnosed with it. Chemotherapy or immunotherapy is the current standard of care for patients with advanced malignant pleural mesothelioma. Cancer drugs that specifically target genetic alterations in mesothelioma have not yet been approved. In this clinical review, we discuss the promising molecular targets and progress made in precision medicine in malignant pleural mesothelioma.

**Abstract:**

Malignant pleural mesothelioma (MPM) is a heterogeneous cancer composed of distinct molecular and pathologic subtypes. Unfortunately, MPM is aggressive, and current therapies for advanced, unresectable disease remain limited to cytotoxic chemotherapy and immunotherapy. Our understanding of the genomic landscape of MPM is steadily growing, while the discovery of effective targeted therapies in MPM has advanced more slowly than in other solid tumors. Given the prevalence of alterations in tumor suppressor genes in MPM, it has been challenging to identify actionable targets. However, efforts to characterize the genetic signatures in MPM over the last decade have led to a range of novel targeted therapeutics entering early-phase clinical trials. In this review, we discuss the advancements made thus far in targeted systemic therapies in MPM and the future direction of targeted strategies in patients with advanced MPM.

## 1. Introduction

Malignant pleural mesothelioma (MPM) is an aggressive cancer composed of malignant mesothelial cells arising from the pleural cavity. MPMs are distinguished by their pathologic subtypes, which include epithelioid, sarcomatoid, and biphasic variants. Histology and stage serve as major prognostic factors in MPM [1]. Epithelial mesotheliomas constitute the most prevalent form of MPM and are associated with a superior prognosis compared to non-epithelial subtypes [2]. Surgery with or without induction or adjuvant chemotherapy is generally reserved for patients with surgically resectable epithelioid-type MPMs, as studies have not shown a survival benefit for surgery in patients with sarcomatoid mesotheliomas [1]. However, the majority of MPM cases present with advanced disease at diagnosis and are, therefore, considered surgically unresectable.

Historically, MPM had a poor prognosis with a 5-year overall survival (OS) of 5% [3]. Systemic therapy with platinum-based chemotherapy and pemetrexed has been the mainstay treatment for unresectable MPM for the last 20 years. In October 2020, the combination of anti-programmed cell death 1 (PD-1) antibody nivolumab and anti-cytotoxic T-lymphocyte 4 (CTLA-4) antibody ipilimumab received approval for use as a first-line treatment for unresectable MPM based on results from CheckMate 743. CheckMate 743 changed the treatment landscape for MPM by establishing a role for immune-checkpoint inhibitors. While nivolumab plus ipilimumab improved OS for all patients with unresectable MPM compared to chemotherapy, subgroup analysis suggests a greater OS benefit with nivolumab plus ipilimumab for patients with non-epithelioid histology [4].

Unlike non-small-cell lung cancer (NSCLC), where significant advances have been made in developing effective targeted therapies, selective agents to treat advanced MPM are still lacking. Nevertheless, as our understanding of the molecular tumorigenesis of MPM continues to evolve, clinical trials are underway to evaluate the efficacy and safety of targeted therapeutics against MPM. In this article, we review the progress that has been made so far in understanding the genomic alteration of MPM and the ongoing efforts to find novel targets to treat this aggressive disease.

## 2. Mesothelin

Mesothelin is a cell-surface glycoprotein that is overexpressed in several tumor types including mesothelioma, as well as ovarian and pancreatic adenocarcinoma [5]. Since the expression of mesothelin is generally restricted to malignant mesothelial tissue, mesothelin is an attractive target in MPM [5]. Studies have shown that mesothelin does play a role in MPM tumorigenesis [6,7]. However, the physiological function of mesothelin has not been fully determined, and mesothelin is not essential for normal tissue function or development [8].

Several early efforts to target mesothelin in MPM have included antibody and antitoxin therapies, although the results from these agents have been underwhelming (Table 1) [7,8]. Amatuximab, a monoclonal antibody against mesothelin, combined with cisplatin and pemetrexed, showed a partial response rate of 40% and stable disease rate of 51% in patients with MPM but did not significantly improve progression-free survival (PFS) compared to historical controls (NCT00738582) [9]. LMB-100, an anti-mesothelin antibody–toxin conjugate, meanwhile, did not exhibit clinical efficacy in patients with mesothelin-expressing solid tumors (NCT02317419, NCT02798536) [10]. There were no responses observed in the 20 patients who received LMB-100, and 10 (50%) patients actually had progressive disease. Antitumor activity with single-agent LMB-100 was primarily limited due to the development of neutralizing drug antibodies in the majority of patients [10]. Given the synergistic antitumor effects observed in vivo combining LMB-100 and immune checkpoint inhibitors, a phase 2 study is ongoing to assess the efficacy of LMB-100, followed by pembrolizumab, in patients with malignant mesothelioma (NCT03644550) [11,12].

Another class of drugs targeting mesothelin is antibody–drug conjugates. Anetumab ravtansine is an antibody–drug conjugate comprising an IgG1 anti-mesothelin antibody linked to a tubulin inhibitor (DM4) (NCT02610140) [13]. Compared to vinorelbine, anetumab ravtansine did not show superior median PFS in patients with advanced MPM previously treated with platinum–pemetrexed chemotherapy [13]. Additionally, a phase 2 study evaluated the approach of combining anetumab ravtansine with a PD-1 inhibitor, pembrolizumab, as a subsequent-line treatment in patients with relapsed MPM (NCT03126630) [14]. However, data from this study did not show any statistically significant difference in response rates or median PFS in patients treated with combination anetumab ravtansine and pembrolizumab versus pembrolizumab alone [14]. Similar to amatuximab and LMB-100, antibody–drug conjugates against mesothelin so far have demonstrated limited efficacy in MPM.

Over the years, chimeric antigen receptor (CAR) T cell therapies have been approved for the treatment of various hematologic malignancies. Conversely, the success of CAR T cell therapy in solid tumors has been challenged by off-target toxicities due to its effects on target antigens that are present on both normal and malignant T cells [18]. CAR T cells designed to temporarily express mesothelin via mRNA electrophoresis have been tested in two patients with mesothelin-expressing tumors. One patient with MPM treated with mRNA CAR T cell therapy achieved a partial response of 6 months [19]. Subsequently, second-generation lentiviral-transduced CAR T cell therapy against mesothelin sought to promote the stable expression of CAR. However, the phase 1 study of CAR T cells generated via lentiviral transduction in five patients with pretreated MPM only obtained stable disease at best in four patients at 28 days, while three patients had disease progression at 3 months (NCT02159716) [15]. Moreover, CAR T cell persistence and the degree of tumor infiltration were low for lentiviral-transduced CAR T cells [15]. One strategy to overcome poor T cell tumor trafficking and persistence involved evaluating the intrapleural administration of mesothelin-targeted CAR T cells, followed by pembrolizumab [16]. Preclinical models showing the ability of anti-PD-1 agents to salvage CAR T cell function and promote antitumor response provided the rationale for adding immunotherapy to CAR T cell therapy [16,20]. A phase 1 study evaluating this approach found detectable CAR T cells in the peripheral blood for more than 100 days in 39% of patients. The median OS was 23.9 months. Out of 18 patients, 8 patients had stable disease for at least 6 months and 2 patients had a complete metabolic response (NCT02414269) [16].

Similar to CAR T cell technology, T cell receptor fusion constructs (TRuC) engage with the immune system to enhance antitumor activity. TRuCs comprise one T cell receptor (TCR) subunit attached to a costimulatory domain and integrated into native CD3 complexes via transduction. Upon the recognition of the specific tumor antigen, TRuCs facilitate the activation of the entire TCR independent of HLA stimulation [17]. In vitro, anti-mesothelin TRuC T cells have demonstrated effective cytotoxicity against different cancer cell lines with high mesothelin expression including mesothelioma, NSCLC, and ovarian adenocarcinoma tumor cells. In mesothelioma xenograft models, mesothelin-targeted TRuC (TC-210) T cells showed a more rapid reduction in tumor volume, enhanced infiltration into tumors, and faster activation and greater persistence in the tumor microenvironment compared to mesothelin-targeted CAR T cells [21]. Given this preclinical data, this suggested the potency of mesothelin-targeted TRuC T cells and provided the foundation for further evaluation of TRuC T cells in clinical studies of MPM.

Gavocabtagene autoleucel (gavo-cel; TC-210) is a TRuC containing a human anti-mesothelin antibody fused to a CD3ε subunit and demonstrated encouraging early results in patients with MPM. Interim data from a phase 1/2 study of gavo-cel after lymphodepletion in patients with treatment-refractory MPM showed an ORR of 21%, a median PFS of 5.6, and a median OS of 11.2 months (NCT03907852). Grade 3 or higher pneumonitis and cytokine release syndrome were observed in 0% and 15% of patients at the recommended phase 2 dose (RP2D) [17]. Gavo-cel is the first targeted therapy to demonstrate tumor response in MPM. However, concerns regarding T cell exhaustion remain, and the phase 2 portion of the trial is ongoing to evaluate the safety and efficacy of gavo-cel as a monotherapy and in combination with immune checkpoint inhibitors.

Advances in T cell therapy have further built upon TRuC T cells by genetically engineering and integrating the expression of chimeric switch receptors (CSRs). Anti-mesothelin TRuC T cells expressing PD-1/CD28 CSRs were developed with efforts to augment and sustain the stimulatory action of anti-mesothelin TRuCs [22]. By simultaneously inhibiting the immunosuppressive effects of PD-L1 signaling and promoting full TCR activation through the costimulatory action of CD28, anti-mesothelin TRuC T cells with PD-1/CD28 CSRs are promising antitumor agents in MPM. Anti-mesothelin TRuC T cells (TC-510) showed improvement in survival and expansion in vitro upon tumor rechallenge compared to TC-210. In vivo, TC-510 demonstrated durable antitumor activity in xenograft models, with less tumor recurrence in mice rechallenged with mesothelioma tumors than those treated with TC-210 [22]. Anti-mesothelin TRuC T cells with PD-1/CD28 CSRs are currently under investigation in a phase 1/2 clinical trial in patients with advanced mesothelin-expressing solid tumors (NCT05451849).

Furthermore, there are a number of early-phase trials assessing other mesothelin-targeted therapies in progress. HPN536 is a T cell engager or TriTAC (Tri-Specific T cell Activating Construct) composed of an anti-mesothelin antibody domain, a single-chain variable fragment against CD3 on T cells, and an anti-albumin antibody domain that prolongs drugs’ half-lives. HPN536 is currently being studied in a phase 1/2a clinical trial in patients with MPM who progressed after first-line platinum-based chemotherapy (NCT03872206) [23]. A phase 1 study is evaluating AMG-994, an anti-mesothelin bispecific antibody, as a monotherapy and in combination with AMG 404, an anti-PD-1 inhibitor, in patients with relapsed or refractory solid tumors expressing mesothelin (NCT04727554). Other phase 1 trials are investigating agents such as a mesothelin-targeted thorium-227 conjugate (NCT03507452) and mesothelin-targeted CAR T cells armored with the IFN-γ-activated secretion of the PD-1 nanobody (NCT05373147).

## 3. BAP1

BRCA1-associated protein 1 (BAP1) is a nuclear deubiquitylase and functions as a tumor suppressor in MPM. *BAP1* is encoded on chromosome 3p21.3 and is among the most common gene alterations in MPM [24]. A majority of *BAP1* mutations in MPM are somatic (70–80%), whereas germline *BAP1* mutations in MPMs are less common and less aggressive than those in MPMs harboring somatic *BAP1* mutations [25]. One feature of BAP1 is its association with BRCA1-associated RING domain 1 (BARD1) and BRCA1, forming the BRCA1-BARD1-BAP1 complex. BRCA1-BARD1-BAP1 acts to mediate homologous recombination repair of DNA double-strand breaks, and this led studies to evaluate the clinical utility of poly (ADP-ribose) polymerase (PARP) inhibitors in MPM (Table 2). However, the phase 2 trial evaluating olaparib in 23 patients with refractory pleural and peritoneal MPM demonstrated limited efficacy, with a partial response in 1 (4%) patient and stable disease in 18 (78%) patients. Median PFS and OS were 3.6 months and 8.7 months, respectively (NCT03531840). Eight (42%) patients had somatic *BAP1* mutations, while four (17%) patients had germline *BAP1* mutations. All patients with germline *BAP1* mutations experienced primary progression on olaparib. Only one patient who harbored a somatic *BAP1* mutation as well as a germline *MRE11A* mutation responded to treatment with olaparib [26]. MRE11 is involved in the resumption of damaged DNA replication and recruited to stall replication forks by PARP [27]. At this juncture, the clinical implications of *MRE11* mutations in MPM require further elucidation.

Rucaparib is another PARP inhibitor that was studied in the phase 2 MiST1 trial (NCT03654833). MiST1 enrolled 26 patients with BAP1- or BRCA1-deficient mesotheliomas. In this study, 10 (38%) patients were BAP1- and BRCA1-negative, 23 (89%) patients were BAP1-negative, and 13 (50%) patients were BRCA1-negative. Rucaparib achieved a disease control rate of 58% at 12 weeks and 23% at 24 weeks. ORR was 12% for patients treated with rucaparib. Based on a post hoc analysis, rucaparib showed a median PFS of 17.9 weeks and OS of 41.4 weeks. Losses of BAP1 or BRCA1 were not predictive of response to rucaparib [28]. Similar to olaparib, rucaparib demonstrated only modest antitumor efficacy in patients with mesothelioma.

BAP1 functions in multiple signaling pathways, another one of which includes regulating the expression of polycomb repressive complex 2 (PRC2) proteins. The enhancer of zeste-homolog 2 (EZH2) is a histone methyltransferase that catalyzes PRC2 and has been found to be overexpressed in 85% of MPMs [29,30]. The inhibition of EZH2 was associated with the decreased proliferation and migration of mesothelial cells in vitro, as well as decreased tumor volume in MPM xenografts in vivo [29]. One study further showed that EZH2 inhibition resulted in reduced tumor size in mice with BAP1-inactivated mesotheliomas compared to wild-type mesotheliomas, providing evidence for the potential role of BAP1 in mediating tumor sensitivity to EZH2 inhibition [31].

Given preclinical data demonstrating the antitumor activity of EZH2 inhibition in *BAP1* mutant mesothelioma, a phase 2 clinical trial evaluated the safety and efficacy of tazemetostat, a selective oral EZH2 inhibitor, in patients with relapsed or refractory MPM (NCT02860286) [31,32]. Seventy-four patients were enrolled in this study, and 73 (99%) patients were confirmed to have *BAP1*-deficient tumors. Despite an ORR of 3%, the disease control rate was 51% at 12 weeks and 28% at 24 weeks. The median PFS was 18 weeks and median OS was 36 weeks. Hyperglycemia, hyponatremia, and anemia were the most common grade 3–4 treatment-emergent adverse events (TEAEs), and serious adverse events occurred in 34% of patients [32]. Data from this clinical study suggest EZH2 inhibition as a potential target for therapeutic intervention in MPMs.

However, the depth and duration of response in MPM with EZH2 inhibition remains suboptimal. The exposure of MPM cells to tazemetostat in vitro has been shown to enhance the recruitment and differentiation of monocytes to a phenotype resembling tumor-associated macrophages (TAMs). In a multicellular spheroid model containing MPM cells treated with tazemetostat, TAM-like monocytes were found to exert tumorigenic and immunosuppressive properties and diminished the antitumor effects of tazemetostat [33]. As a result, the presence of these TAM-like monocytes poses a mechanism of resistance to tazemetostat and suggests that combination therapies against EZH2 and TAMs may offer greater antitumor efficacy [33].

**Table 2:** Molecular targets in malignant pleural mesothelioma.

Tumor Suppressor	Prevalence in MPM	Clinical Trial	Drug	Synthetic Target	Efficacy
BAP1	70–80% [25]	NCT03531840	Olaparib	PARP	ORR, 4%; SD, 78%; median PFS, 3.6 months; median OS, 8.7 months [26]
		NCT03654833	Rucaparib	PARP	DCR, 58% at 12 weeks; ORR, 12%; median PFS, 17.9 weeks; median OS, 41.4 weeks [28]
		NCT02860286	Tazemetostat	EZH2	DCR, 51% at 12 weeks; ORR, 3%; median PFS, 18 weeks; median OS, 36 weeks [32]
*NF2*	35–40% [34,35]	NCT04665206	VT3989	YAP/TEAD	ORR, 14% [36]
		NCT04857372	IAG933	YAP/TEAD	-
*CDKN2A*	60–74% [37,38,39,40]	NCT03654833	Abemaciclib	CDK4/6	DCR, 54% at 12 weeks; ORR, 12%; median PFS, 128 days; median OS, 217 days [41]
*MTAP*	67% [42]	NCT05275478	TNG908	PRMT5	-
		NCT05245500	MRTX1719	PRMT5	-
AXL	75% [43]	NCT03654833	Bemcentinib + pembrolizumab	-	DCR, 46.2% at 12 weeks; ORR, 15.4% [44]
ASS1	48–63% [45,46]	NCT01279967	ADI-PEG20 (First-line or subsequent therapy)	Arginine deiminase	SD, 52% at 4 months; ORR, 0%; median PFS, 3.2 months; median OS, 11.5 months [46]
		NCT02709512	ADI-PEG20 (First-line therapy)	Arginine deiminase	ORR, 13.8%; median PFS, 6.2 months; median OS, 9.3 months [47]

Abbreviations: ASS1, arginine succinate synthetase 1; AXL, anexelekto; BAP1, BRCA1-associated protein 1; *CDKN2A*, cyclin-dependent kinase inhibitor 2A; DCR, disease control rate; EZH2, enhancer zeste homolog 2; *MTAP*, methylthioadenosine phosphorylase; *NF2*, neurofibromatosis type 2; ORR, objective response rate; OS, overall survival; PFS, progression-free survival; SD, stable disease.

## 4. NF2

*NF2* encodes for merlin, a tumor suppressor and member of the moesin–ezrin–radixin family of membrane-cytoskeleton proteins [48]. Merlin is an upstream regulator of multiple signaling pathways including the Hippo cascade. Approximately 35–40% of MPMs carry inactivating *NF2* mutations, resulting in the inactivation of the Hippo pathway and dephosphorylation and activation of downstream transcriptional cofactors, YAP1, yes-associated protein 1, and TAZ, a transcriptional coactivator with PDZ-binding motif [34,35]. YAP1/TAZ interact with TEAD, a transcriptional-enhancer-associated domain, transcription factors to form an activating complex that promotes the gene transcription of proteins for cell proliferation and survival [34]. Inhibiting the YAP/TEAD interaction is a promising therapeutic opportunity for preclinical data that demonstrated tumor regression in mesothelioma xenograft models with TEAD palmitoylation inhibition [34].

VT3989 is an oral inhibitor of TEAD palmitoylation and the first in its class to target YAP/TEAD activation. Antitumor activity with VT3989 has been observed in patients with mesothelioma, with data from a phase 1 study demonstrating the clinical feasibility of selectively inhibiting Hippo signaling in mesothelioma (NCT04665206). Of the 43 (62%) patients with malignant mesothelioma who received VT3989, 6 (14%) patients had a partial response, and the clinical benefit response at 8 weeks was 57% [36]. For three patients with responses to VT3989, the duration of the response was at least 21 months [49]. Furthermore, this phase 1 trial suggested the safety of VT3989 as there were no reported dose-limiting toxicities. Albuminuria, peripheral edema, fatigue, and nausea were the most common side effects of VT3989 [36].

As a class, YAP/TEAD inhibitors are novel agents currently being examined in early-phase clinical trials. Another YAP/TEAD inhibitor is IAG933, a small molecule that disrupts the YAP/TAZ-TEAD interface [50]. IAG933 has entered a phase 1 trial in patients with previously treated advanced mesothelioma, *NF2/LATS1/LATS2* mutated tumors, and YAP/TAZ fusion-positive tumors (NCT04857372).

## 5. CDKN2A

Homozygous deletion in *CDKN2A* occurs in 60–74% of MPMs and represents the most common genetic alteration in MPMs. *CDKN2A* encodes for p16ink4A, a tumor suppressor that inhibits cyclin-dependent kinases (CDK) 4 and 6 and their presence in patients with MPM is associated with inferior survival outcomes [37,38,39,40]. CDK4/6 inhibitors, abemaciclib and palbociclib, showed efficacy in preclinical studies, with evidence of decreased cell proliferation and induction of cell senescence in MPM cells exposed to either drug [51,52]. In vivo, palbociclib resulted in reduced tumor volume and improved survival compared to chemotherapy in MPM mouse models [51].

Given the antitumor activity of CDK4/6 inhibitors on preclinical models of MPM cell lines and xenografts, a phase 2 clinical trial, MiST2, sought to test the clinical efficacy of abemaciclib in patients with p16ink4A-negative mesothelioma previously treated with platinum chemotherapy (NCT03654833). All patients enrolled in MiST2 had MPM, and out of 26 patients in this study, 14 (54%) patients achieved disease control at 12 weeks. In total, 3 (12%) patients obtained a partial response, and 11 (42%) patients had stable disease [53]. Since *MTAP* is frequently codeleted with *CDKN2A* due to its proximity to *CDKN2A* on the same gene locus, a post hoc analysis found 11 (44%) patients with loss of both *MTAP* and *CDKN2A* expression. Moreover, there was a greater reduction in tumor volume observed in patients with *MTAP*-negative mesotheliomas compared to those with *MTAP*-positive mesotheliomas, suggesting that *CDKN2A* and *MTAP* codeletions may select for patients who will derive a greater benefit from abemaciclib [53]. As for the safety profile of abemaciclib, fatigue, diarrhea, nausea, decreased appetite, and anemia were the most common side effects [53].

MiST2 was the first clinical study to show the antitumor activity of a CDK4/6 inhibitor as a single agent in patients with MPM. Combination therapies that include targeting *CDKN2A* inhibition could offer opportunities for enhancing treatment efficacy. One study showed reduced cell proliferation in MPM cells treated with abemaciclib plus cisplatin and pemetrexed versus chemotherapy alone [41]. Abemaciclib plus chemotherapy also induced more durable antiproliferative effects in vitro, which suggests that the addition of abemaciclib may play a role in overcoming resistance to chemotherapy [41]. Given these preclinical data, combining CDK 4/6 inhibitors and chemotherapy is a potential therapeutic strategy and requires further evaluation in prospective clinical trials.

## 6. MTAP

*MTAP* deletions are detected in two thirds of MPMs and occur as codeletions with *CDKN2A* in the majority of pleural mesotheliomas [42]. *MTAP* encodes for methylthioadenosine phosphorylase, an enzyme that converts methylthioadenosine (MTA) into metabolites for AMP and methionine synthesis [42]. Homozygous *MTAP* deletions result in MTA accumulation, and this, in turn, inhibits protein arginine methyltransferase 5 (PRMT5) catalytic activity [54]. Consequently, the formation of PRMT5-MTA complexes sensitizes *MTAP*-deleted cancer cells to enable further PRMT5 inhibition [54]. PRMT5, therefore, can serve as a synthetic lethal target for *MTAP*-deleted tumors.

On the basis of preclinical evidence showing tumor regression in *MTAP*-deleted xenograft models with PRMT5 inhibition, a phase 1/2 study is currently recruiting patients with locally advanced or metastatic *MTAP*-deleted mesothelioma to receive TNG908, a selective PRMT5 inhibitor in *MTAP*-null cancers (NCT05275478) [55]. Additionally, a phase 1/2 trial of MRTX1719, a small molecule that selectively binds to the PRMT5/MTA complex to block PRMT5, is enrolling patients with advanced solid tumors with *MTAP* deletion (NCT05245500). Early clinical data regarding MRTX1719 show activity across different tumor types, with responses seen in patients with *MTAP*-deleted pleural mesothelioma, melanoma, gallbladder adenocarcinoma, NSCLC, and malignant peripheral nerve sheath tumors [56].

## 7. AXL

Anexelekto (AXL) is a member of the TAM (Tyro3, AXL, Mer) family of receptor tyrosine kinases that binds to Gas-6 to activate the RAS/RAF/MAPK and PI3K/AKT/S6K signaling pathways [43]. The activation of AXL promotes cell survival, growth, and invasion, as well as resistance to immunotherapy [57]. AXL mediates immune escape by restricting pro-inflammatory cytokine expression through the upregulation of BCL-2 and Twist and suppressing inflammatory Toll-like receptor signaling and natural killer cell activity [57,58]. AXL overexpression is found in 75% of MPMs and is associated with poor survival outcomes [43,59].

MiST3 was a phase 2 study that evaluated the efficacy of a combination of bemcentinib, a selective AXL tyrosine kinase inhibitor, and pembrolizumab in patients with previously treated MPM (NCT03654833). A majority of patients enrolled in this trial had relapsed epithelioid MPM (88%) compared to biphasic (8%) and sarcomatoid (4%) histologies. The disease control rate at 12 weeks was the primary endpoint, and it was 46.2% in this trial. The ORR was 15.4%, and the disease control rate at 24 weeks was 38.5%. Fatigue and nausea were the most common side effects of bemcentinib plus pembrolizumab [44]. Data regarding the molecular indicators of response are yet to be published and may help us to select patients who are most likely to derive clinical benefit from this combination therapy.

## 8. ASS1

Arginine succinate synthetase 1 (ASS1) is a urea cycle enzyme involved in the synthesis of arginine, an amino acid essential for cell survival and growth. ASS1 deficiency is found in 48–63% of MPMs and occurs predominantly in the sarcomatoid and biphasic MPM subtypes [45,46]. Exogenous arginine is crucial for ASS1-deficient cancers, and malignant mesothelioma has an auxotrophic dependency on arginine in which exogenous arginine deprivation induces apoptosis of malignant mesotheliomas cells with ASS1 loss [45]. Hence, there has been interest in examining the clinical impact of therapeutics that exploit arginine depletion in MPM.

A multicenter phase 2 randomized study (ADAM) of pegylated arginine deiminase (ADI-PEG20) in treatment-naïve and pretreated patients with ASS1-deficient MPM showed a median PFS of 3.2 months with ADI-PEG20 versus 2.0 months with best-supportive care (*p* = 0.03; HR 0.56) (NCT01279967). At 4 months, stable disease was the best response and occurred in 52% of patients in the ADI-PEG20 group, compared to 22% in the best-supportive care group [46]. ADI-PEG20 was further studied in the phase 2/3 ATOMIC-meso trial as a first-line therapy in combination with platinum chemotherapy plus pemetrexed versus chemotherapy alone in 249 patients with non-epithelioid MPM (NCT02709512). A superior median OS of 9.3 versus 7.7 months was observed for the ADI-PEG20 combination arm. The median PFS was slightly improved at 6.2 versus 5.6 months with the addition of ADI-PEG20, and ORR was similar for both groups at 13% [47]. ADI-PEG20 and chemotherapy may be a viable option for patients with non-epithelioid MPM, but this combination would need to be directly compared to treatment with dual checkpoint inhibitors as the median OS was numerically higher at 18.1 months with nivolumab plus ipilimumab in CheckMate 743 [4,60].

## 9. Conclusions

MPM is an aggressive cancer, and its molecular signature is characterized by inactivating alterations in tumor suppressor genes, resulting in the dysregulation of multiple intracellular signaling pathways and tumorigenesis [61]. Contrary to some solid tumors, where the presence of oncogene-activating mutations portends therapeutic sensitivity to targeted tyrosine kinase inhibitors (TKIs), the molecular landscape of MPM has generally been considered to lack such genetic driver mutations. The overexpression of epidermal growth factor receptor (EGFR), EphB4, MET, and AXL have been described in MPM and implicated in MPM oncogenesis [62,63,64,65]. However, phase 2 studies that evaluated the efficacy of two EGFR TKIs, erlotinib and gefitinib, as first-line therapeutics in patients with MPM did not demonstrate clinical activity with EGFR TKIs [66,67]. Although there is preclinical evidence for greater antitumor activity with combination strategies against MET and AXL signaling, this needs to be confirmed in prospective clinical studies [68,69].

Applying synthetic lethality to drug development in MPM is a novel approach that has opened avenues for targeting previously undruggable pathways. Synthetic lethality is the concept of targeting a single ‘partner’ protein in the setting of a coexisting mutation to impart an antitumor response [70]. Strategies that aim to take advantage of synthetic lethality in MPM are promising as MiST2 showed clinical activity with CDK4/6 inhibition in *CDKN2A*-deleted mesothelioma, and clinical trials are ongoing to assess the effectiveness of YAP/TEAD and PRMT5 inhibitors in *NF2-* and *MTAP*-mutated MPM, respectively.

As knowledge of the molecular characteristics of MPM continues expanding, delineating the relationship between these genomic aberrations and the tumor microenvironment may offer insight into novel strategies for combining targeted therapies with immune checkpoint inhibitors. For example, mouse models of breast cancer and other solid tumors have shown that exposure to CDK4/6 blockade can promote T cell-mediated antitumor activity such that this may be further amplified via immune checkpoint inhibition [71,72]. As discussed earlier in this review, MiST3 suggests that AXL inhibition and pembrolizumab can act synergistically to confer antitumor activity [44]. Despite the low tumor mutation burden of MPM, CheckMate 743 established a role for immunotherapy in MPM, and the effect of combining targeted therapy and immune checkpoint inhibitors in select patients with specific genomic alterations warrants further evaluation [73].

At this time, there are no targeted therapeutics approved for the treatment of MPM. Although preclinical data have provided evidence for a host of potential therapeutic targets, other factors such as molecular and pathologic features are important in ascertaining which subset of patients will attain the greatest clinical response from targeted therapies. Hence, moving the needle in precision medicine in MPM requires ongoing research to identify predictive biomarkers that will help to stratify patients to the most effective targeted strategy.

## Figures and Tables

**Table 1:** Mesothelin targeted therapies in malignant pleural mesothelioma.

Drug Class	Drug	Clinical Trial	Efficacy
Antibody	Amatuximab	NCT00738582	ORR, 40%; SD, 51%; median PFS, 6.1 months; median OS, 14.8 months [9]
Antibody–toxin conjugate	LMB-100	NCT02317419 NCT02798536	ORR, 0%; median PFS, 2.8 months; PD in all patients within 3 months of starting LMB-100 [10]
	LMB-100 plus pembrolizumab	NCT03644550	-
Antibody–drug conjugate	Anetumab ravtansine	NCT02610140	ORR, 9.6%; DCR, 73.5%; median PFS, 4.3 months; median OS, 9.5 months [13]
	Anetumab ravtansine plus pembrolizumab	NCT03126630	ORR, 11%; SD, 50%; median PFS, 12.2 months [14]
CAR T cell	CART-meso cell	NCT02159716	PD in 3 out of 5 patients within 3 months of receiving CART-meso cell [15]
	huCART-meso cell	NCT03054298	-
	Intrapleural delivery of CAR T cells followed by pembrolizumab	NCT02414269	ORR, 12.5%; SD, 56.3%; median PFS, 23.9 months [16]
	αPD1-MSLN-CAR T cell	NCT05373147	-
T cell receptor fusion construct (TRuC)	Gavocabtagene autoleucel (TC-210)	NCT03907852	ORR, 21%; median PFS, 5.6 months; median OS, 11.2 months [17]
	TC-510	NCT05451849	-
Tri-specific T cell activating construct	HPN536	NCT03872206	-
Bispecific antibody	AMG-994	NCT04727554	-
Thorium-227-labeled antibody–chelator conjugate	BAY2287411	NCT03507452	-

Abbreviations: CAR, chimeric antigen receptor; DCR, disease control rate; MSLN, mesothelin; ORR, objective response rate; OS, overall survival; PD, progression of disease; PD1, programmed cell death protein 1; PFS, progression-free survival; SD, stable disease; TRuC, T cell receptor fusion construct.
